# Reversible Thermochromic Nanocomposites Based on Thiolate-Capped Silver Nanoparticles Embedded in Amorphous Polystyrene

**DOI:** 10.3390/ma2031323

**Published:** 2009-09-18

**Authors:** Gianfranco Carotenuto, Francesca Nicolais

**Affiliations:** 1 Institute of Composite and Biomedical Materials, National Research Council, Piazzale Tecchio, 80 – 80125 Napoli, Italy; 2 Department of Communication Sciences, University of Salerno, Via Ponte don Melillo – 84084 Fisciano, Salerno, Italy; E-Mail: franicolais@gmail.com (F.N.)

**Keywords:** thermochromism, thiolate-capped silver nanoparticles, interdigitation, surface plasmon absorption

## Abstract

Technologically useful reversible thermochromic materials can be prepared using very simple polymer-embedded nanostructures. In particular, silver nanoparticles capped by long-chain alkyl-thiolate molecules (i.e., Ag_x_(SC_n_H_2n+1_)_y_, with n > 10) spontaneously organize in aggregates because of the interdigitation phenomenon involving the linear alkyl chains bonded at surfaces of neighboring nanoparticles. Owing to the alkyl-chain interdigitation, nanoparticles very close to each other result and an interaction among their surface plasmon resonances may take place. Surface plasmon interaction causes a splitting of the absorption band whose characteristics depend on the aggregate shape. Since shape-less aggregates are generated, a multiple-splitting of the silver surface plasmon absorption band is observed, which causes a broad absorption spreading on the whole visible spectral region. Amorphous polystyrene containing interdigitated silver nanoparticles has a dark-brown or black coloration, depending on the nanoparticle numerical density, but since the inter-particle distance slightly increases at melting point of interdigitation crystallites a reversible termochromic effect is observed at this special temperature. In particular, the material coloration changes from dark-brown to yellow which is the coloration produced by the surface plasmon absorption of isolated silver nanoparticles. This reversible thermochromism can be finely controlled by modifying the structure of thiolate groups, and precisely, the strength of interactions acting inside the interdigitation crystallites.

## 1. Introduction to Nanocomposite Thermochromism

The properties of metals and semiconductors mainly depend on their electronic configuration, which results significantly modified when they are reduced to a nanoscopic scale. Consequently, these solids may show anomalous physical behaviors as their size approaches to a few nanometers [1,2,3,4,5], and new optical, magnetic, electronic, transport, etc. properties can be observed. Such novel type of solid matter is named mesoscopic matter, and the physical properties of solids are not anymore those of bulk-solids (massive matter) and not yet those of the constituting atoms or molecules in this particular size regime.

**Figure 1:**
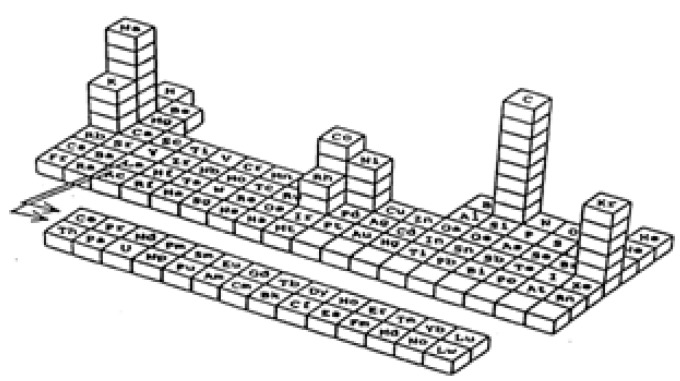
3D-Periodic table of elements.

The mesoscopic regime extends over a dimensional range which is depending on the type of phenomenon under consideration, and it may range from 1 nm to 100 nm for certain phenomena, or be very close to only one nanometers for others. For example, to observe luminescence in metallic phases a size of a few nanometers is required (atomic metal nanoparticles) [6,7,8], while the characteristic coloration of nanoscopic gold particles (light absorption by surface plasmon resonance) appears at sizes of several nanometer tens [5]. Analogously, super-catalytic properties are visible only for noble-metal crystals of extremely small sizes [9], while ferromagnetic particles becomes super-paramagnetic at a dimension approaching that of the single magnetic domain which is usually of several nanometer tens [10]. Since the same type of element may show a different set of physical, chemical, thermodynamic, catalytic, etc. properties depending on the size, a 3D-periodic table of elements has been proposed (see Figure 1) [11].

Nanoscopic solids can be used in an embedded form and polymers represent a very convenient type of matrix. Usually, these nanostructured materials are named nanocomposites because they are morphologically similar to the discontinuous polymeric composites [12,13,14,15,16,17].

A conveniently selected polymer containing nanoscopic metal or semiconductor particles may show some of the physical characteristics of the hosted phase. A functional material whose properties are based on some anomalous physical behavior of matter at mesoscopic regime represent a ‘mesoscopic device’ [12]. Examples of mesoscopic devices for optical applications are color filters and other types of optical limiters (e.g., UV-absorbers, IR-filters, etc.), based on the surface plasmon resonance of nanoscopic coin metals like silver, gold, copper and their alloys (alloying makes possible to control the density of electrons in the conduction band and therefore to tune the frequency of surface plasmon resonance). Also fluorescent plastics based on metallic quantum-dots confined into a dielectric optical plastic represent a technologically important type of mesoscopic device, and analogously super-paramagnetic optical plastics consisting in polymer-embedded nanoscopic metal particles with magnetic properties. Such mesoscopic devices which combine together high transparency in the visible spectral range to some novel property, coming from the nanoscopic size of the embedded inorganic phase are potentially exploitable in different technological fields. For example, super-paramagnetic optical plastics can be used in magneto-optics, transparent-fluorescent plastics are useful for photonic modulators, etc.

Therefore, metal and semiconductor nanoparticles can be used as building-blocks of a mesoscopic device. Polymer-embedding prevents naked-nanoparticles from aggregation and self-assembly, however these nanoparticles are quite unstable also in a polymer-embedded form because of surface oxidation and/or contamination phenomena. Small molecules like oxygen, water, SO_2_ may diffuse into the polymer matrix, modifying the chemical nature of the metal surface and therefore affecting significantly the functional properties of the nanoparticle system. Actually, also noble-metal nanoparticles can be chemically modified at surface owing to the enhanced chemical reactivity of nanoscopic metals. Usually, a chemical passivation treatment of the metal nanoparticle surface is the only way to achieve stable functional properties for these systems. Transition metal nanoparticles are high electrophilic species owing to the presence of empty *d*- and *f*-orbitals of the surface atoms, and therefore they may absorb nucleophilic ligand molecules on their surface, leaving to nanoparticle compounds. For example, strong ligand molecules like phosphines (e.g., PR_3_, where R is an alkyl group) can be stably bonded to the metal nanoparticle surface leaving to air-stable nanoparticle compounds.

However, thiolate-capping is a very common chemical passivation treatment for noble-metal nanoparticles, like for example the thiolate-capped gold nanoparticles, named thioaurites [18]. Thioaurites are typical nanoparticle compounds, they are derived from the reaction of naked gold-nanoparticles with thiol molecules, R-SH. Such reaction takes place according to the following scheme:

Au_n_ + m RSH → Au_n_(SR)_m_ + m/2 H_2_ (1)

**Figure 2:**
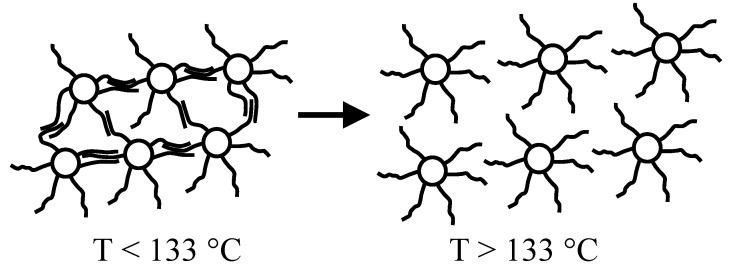
Schematic representation of the crystallization by interdigitation for nano-particles of silver capped by dodecyl-thiolate.

In a similar way, naked nanoparticles of silver and other noble-metals (e.g., Cu_n_, Pt_n_, Pd_n_, etc.) may give thiol-derivatized nanoparticles that are much more stable than the starting structures [5]. Nanoparticle compounds are chemically inert species, quite soluble in non-polar organic solvents, and if fractioned up to achieve a monodispersed system, they spontaneously organize in 2D- and 3D-superlattices by self-assembly [18]. Metal nanoparticles capped by linear thiolate molecules (C_n_H_2n+1_-SH), containing a number of carbon atoms higher than 10 have an important physical characteristic, consisting in the co-crystallization of alkyl chains present on the surface of neighbor nanoparticles by interdigitation (see Figure 2). The melting of such crystallites takes place at quite mild temperature values (close to 100 °C), and it is characterized by an enthalpy variation that can be accurately measured by a calorimetric approach (i.e., Differential Scanning Calorimetry, DSC) [19,20].

Therefore, stable optical devices based on polymer-embedded nanoscopic metal phases can be made by using thiolate-capped noble-metal nanoparticles. In this case, nanoparticles do not undergo oxidation/surface-contamination phenomena and consequently the resulting devices may show constant physical properties in service. Depending on the type of capping thiolate molecules used (long chain or short chain), two different topologies are possible for such systems: (i) nanoparticles may form a contact-free dispersion when capped by short-chain thiolate, and (ii) nanoparticles may leave to small coagulation aggregates when coated by long-chain thiolate. In the first case, nanoparticles are perfectly distributed inside the embedding dielectric matrix and an uniform inter-particle distance (much larger than the nanoparticle diameter) characterizes this system. In the latter, nanoparticles form small aggregates uniformly dispersed inside the amorphous polymeric matrix, and inside these aggregates, nanoparticles touch each other but their surfaces are separated by the thin thiolate-capping mono-layers and therefore their surface appear located at a distance of approximately twice the thickness of thiolate coating. In particular, this type of nanoparticle aggregates are named ‘coagulation aggregates’ because they are different from the ‘coalescence aggregates’, where the nanoparticle metallic cores result sintered together [21]. When the aggregate average size is small enough, the embedding-polymer transparency leaves unmodified and therefore this material can be used for optical applications just like a polymer containing a contact-free dispersion of nanoparticles. However, amorphous polymers containing larger nanoparticles aggregates are quite opaque owing to the light-scattering phenomena in the visible spectral region produced by the hosted aggregates. Therefore, the film transparency depends on the average aggregate extension, and it reduces significantly with increasing of this parameter [21].

In the case of nanoparticles capped by thiolate molecules and organized in form of aggregated structures, because of thiolate chain interdigitation, the optical properties of the resulting polymer-embedded systems are strongly dependent on the temperature. In fact, the optical properties result significantly modified at melting point of crystallites produced by the interdigitated chains, showing a step-discontinuity closed to this special point.

Therefore, optical devices based on some nanoparticle property can be classified as passive and active devices. In a passive optical device, the functional properties are not depending on external stimuli like temperature, pressure, etc. On the contrary, the functional properties of an active optical device are influenced by the effect of external stimuli like the temperature. For example, an amorphous polymer containing aggregates of thiolate-capped metal nanoparticles represents an example of active device. In fact, if the aggregates have an average size comparable with light wavelength a scattering phenomenon takes place in the film which results opaque. However, at high temperatures (above the melting point of interdigitation crystallites) the aggregates dissolves and diffuse into the polymer matrix, leading to a contact-free topology with large inter-particle distance. This material is transparent to the visible light.

**Figure 3:**
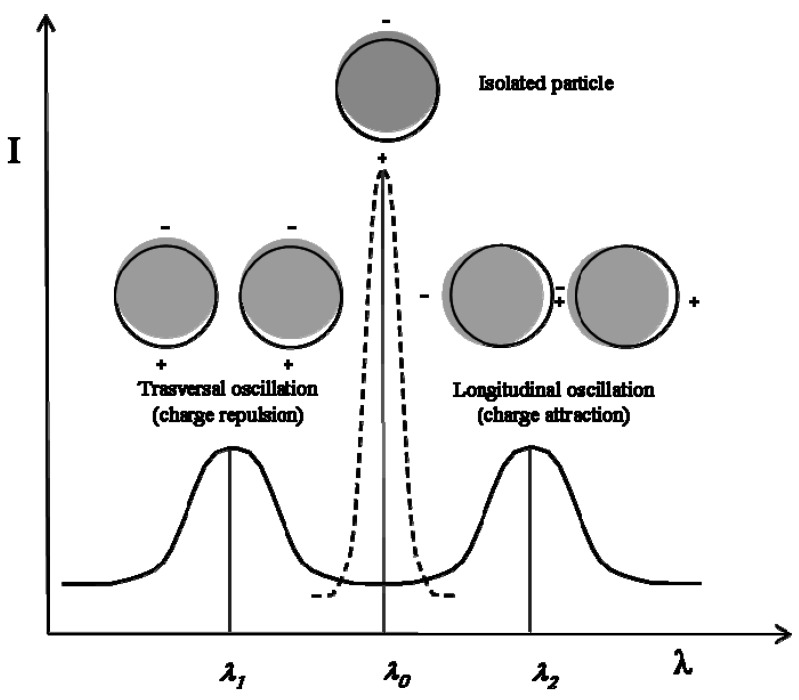
Schematic representation of the splitting in the surface plasmon absorption of a spherical particle caused by the interaction with another particle (actually, the real splitting is not symmetric).

The surface plasmon resonance of a metal nanoparticle system is very sensitive to the inter-particle distance [21], and technologically useful active optical devices can be based on the surface plasmon absorption characterizing the thiolate-capped metal nanoparticles (e.g., silver nanoparticles capped by dodecyl-thiolate) embedded into dielectric polymer matrices. Insulated metal nanoparticles have a single Gaussian surface plasmon absorption band, located at a frequency which is depending on the density of electrons in the conduction band. For example, contact-free silver particles of a few nanometers absorb at ca. 430 nm and the absorption frequency is just slightly modified by the effect of a thiolate-capping layer (this type of nanoparticles produces a bright-yellow coloration in the embedding polymer, leaving unmodified the plastic transparency). However, in the case of nanoparticle couples (the smallest aggregates), these metallic domains are located very close to each other and therefore the charges produced on their surface by the light-induced polarization may interact causing a splitting of the absorption band (see Figure 3). The splitting is observed since the transversal oscillation is impeded, while the longitudinal one is facilitated. In the case of larger aggregates the effect of the near charges is even more intensive and the splitting is depending on the aggregate size and shape (e.g., neck-lace like aggregates, square-shaped aggregates, etc.). In a system of shape-less particle aggregates (aggregates of different shape and size) a multiple splitting process takes place which leaves to a single very broad absorption band. An absorption band extending on the full visible range may produce a black or brown coloration, depending on the uniformity of the absorption intensity (a quite uniform absorption leads to a black coloration, while a non-uniform absorption gives light- or dark-brown absorption).

When a polymeric dispersion of aggregated silver particles transforms to a contact-free particle dispersion by the effect of a temperature increase, the system coloration switches from brown to yellow at melting point of crystallites produced by the interdigitation of thiolate-capping layers. Therefore, a variety of reversible thermochromic plastics can be simply produced by dispersing Ag nanoparticles capped by long-chain thiolates into a polymer. The color switching temperature depends on the melting point of interdigitated crystallites, since at this special temperature the inter-particle distance changes as a consequence of the expansion associated with melting process. Being the color change based on a thermodynamic transition (the melting of crystallites produced by the interdigitated thiolate molecules chemisorbed on the silver nanoparticle surface), the thermochromism is completely reversible, quite prompt and without hysteresis.

## 2. Material Preparation and Characterization

Thermochromic films made of thiolate-capped silver nanoparticles embedded into amorphous polystyrene have been prepared starting from the corresponding silver-thiolate precursors whose characteristics are given in Table 1. The silver(I)-thiolates (AgSR, where R is: CH_3_(CH_2_)_n_- or HO-(CH_2_)_11_-) are chemical compounds not commercially available, and therefore they were obtained by chemical synthesis [22,23]. In particular, these compounds were prepared by adding an acetone solution of thiol, RSH, to a silver nitrate solution (AgNO_3_, Aldrich, 99.9%) in acetonitrile (CH_3_CN, Aldrich, 99.9%) in a dropwise manner at room temperature, under stirring (stoichiometric amounts of reagents were used). An exchange reaction (methatesis) between thiol proton and silver atom took place, according to the following scheme:

AgNO_3_ + RSH → AgSR + HNO_3_ (2)

A white microcrystalline precipitate was immediately produced. The solution was stirred for 24 h at room temperature, then silver(I)-thiolates was separated by vacuum-filtration, washed several times with acetone, and stored in a dry atmosphere until use.

**Figure 4:**
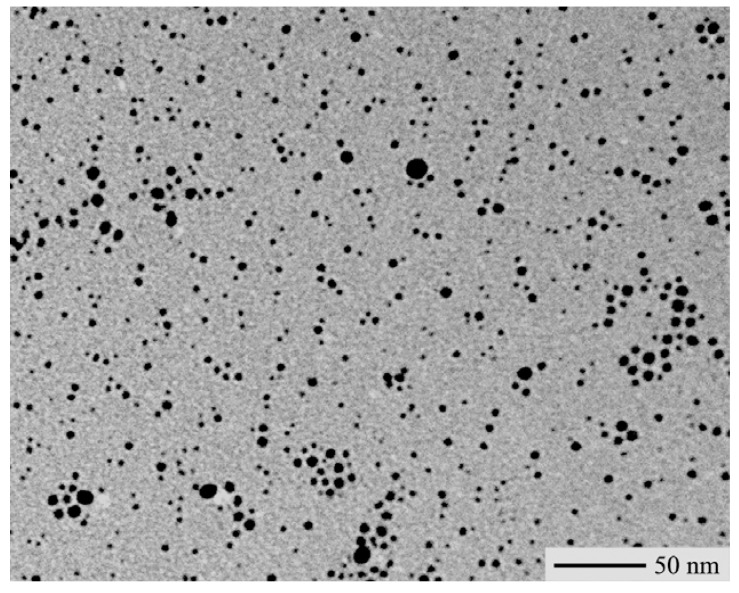
TEM-micrograph of a silver-polystyrene nanocomposite sample obtained by annealing a AgSC_12_H_25_/polystyrene blend.

Homogeneous AgSR/polymer blends were obtained by slowly drying (for two days at room temperature) a mixture of AgSR and polystyrene (PS, Aldrich, M_w_ = 230,000 g·mol^-1^) dissolved in chloroform. All samples were prepared using a 1:10 AgSR/PS weight ratio. The resulting AgSR/polystyrene blends were white colored and opalescent for the presence of AgSR micro-crystals. Then, the blends were isothermally annealed at 200 °C for 3 min using a hot stage for optical microscopy (Mettler). During the AgSR thermolysis stage, a fine dispersion of thiolate-capped silver nanoparticles aggregates in polystyrene were produced [24]. A TEM-micrograph of the silver-polystyrene sample obtained by annealing AgSC_12_H_25_/polystyrene blends is shown in Figure 4, however samples obtained by using other types of silver thiolates exhibited a quite similar morphology. According to the TEM characterization, these films contained spherical silver nanoparticles with an average size of ca. 3 nm organized into shape-less coagulation aggregates with a size of a few nanometer tens.

For this reversible thermochromic material, the best color switching characteristics corresponded to a color change from black (or at least a very dark-brown) to bright-yellow. To produce very dark films the presence of a large amount of aggregates of thiolate-capped silver nanoparticles was required. The formation of these aggregates can be favored by causing the presence of strong concentration gradients inside the precursor silver-thiolate/polymer blend. Such silver-thiolate concentration gradients can be simply generated into the thiolate/polymer blend by a fast evaporation of the solvent (chloroform) during the film casting stage. In this case, the solution was simply put under the air flux of a fume hood, which continuously removed the solvent vapors from the liquid phase surface, allowing a fast system drying.

**Figure 5:**
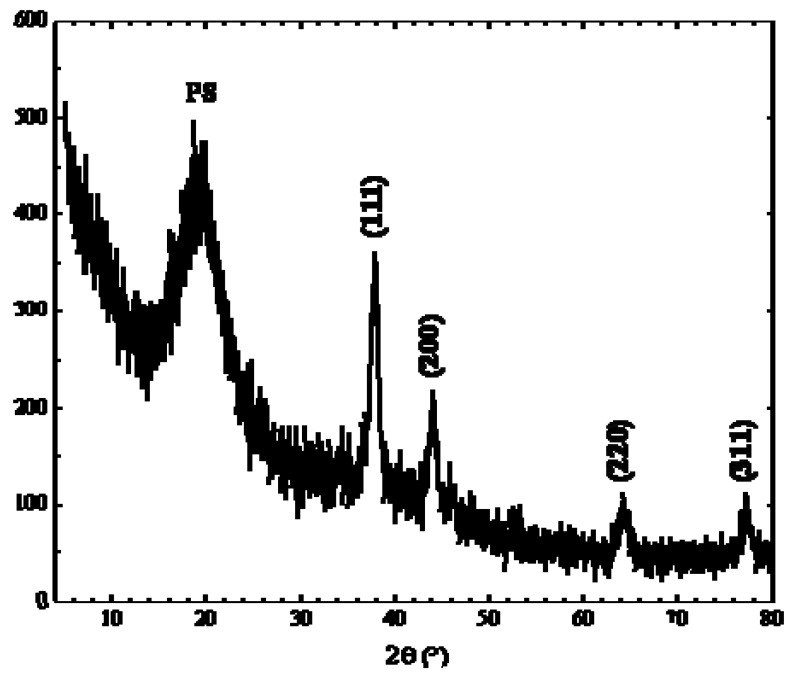
XRD-diffractogram of a silver-polystyrene nanocomposites sample obtained by annealing a AgSC_12_H_25_/polystyrene blend.

In order to understand the crystalline structure of the silver nanoparticles, X-ray diffraction (Rigaku DMAX-IIIC, using Cu-Ka radiation, λ = 1.5418 Å) was carried out to investigate the atomic structure of small nanoparticles. The typical XRD pattern of the as-prepared nanocomposites is shown in Figure 5. As visible, the diffractogram includes four diffraction peaks at 2θ° of 38.4, 44.5, 64.7, and 77.6 corresponding respectively to the [111], [200], [220] and [311] planes of the face-centered cubic (fcc) metallic silver. According to Scherrer’s equation, the silver particle size is ca. 3.5 nm (based on the calculation made using the width of [111] signal), which is consistent with the result of TEM analysis. Absence of the precursor peaks confirmed that the adopted thermal annealing conditions (i.e., annealing time and temperature) allowed complete degradation of thiolate precursor. In addition, the diffraction pattern of silver sulfide, Ag_2_S, (it is produced by a competitive reaction of silver thiolate thermal degradation) was present in very low amount.

The temperature dependence of optical properties of the obtained nanocomposite materials, was determined by measuring the UV-visible absorption spectra of films isothermally heated at growing temperature values. This spectra were measured using a PerkinElmer Lambda 850 spectrometer equipped with a Peltier apparatus (PTP-1, PerkinElmer). In order to analyze the optical spectra in the temperature range 0–200 °C with an accuracy of *±*0.1 °C, the commercial Peltier device was modified by increasing the limit temperature of the hot-stage and including a water refrigeration system. In addition, to completely remove the contribution of polystyrene absorption from the optical spectra, which is quite relevant in the UV region, pure amorphous polystyrene films with a thickness exactly equal to the analyzed thermochromic films were placed in the reference sampling area and heated at the same temperature like the thermochromic samples.

The thermal characteristics of nanocomposite samples (i.e., interdigitated crystals melting range) were evaluated by dynamic tests performed by a Differential Scanning Calorimeter (DSC, Thermal Analyst 2900), calibrated by indium standard. Samples of 7 mg were sealed into hermetic aluminum pans and the DSC tests were done under a purging atmosphere of nitrogen gas from 70 °C to 180 °C at a rate of 10 °C/min for the different thermochromic samples.

## 4. Study of Nanocomposite Thermochromism and Thermal Properties

**Figure 6:**
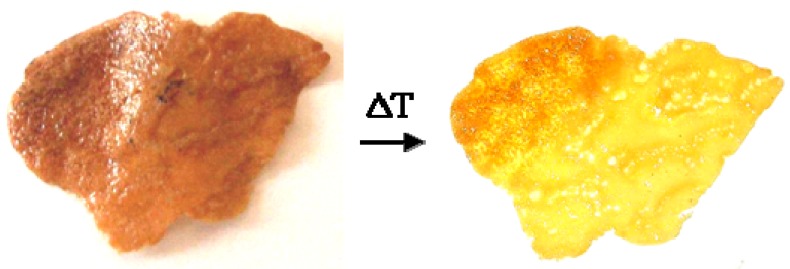
Characteristic reversible thermochromism of a film based on dodecyl-thiolate capped silver nanoparticles embedded into amorphous polystyrene (the material has been thermally cycled for ca. 30 times).

The nanocomposite samples in form of films showed a well-visible thermochromic effect, characterized by a prompt and reversible color change from dark-brown (or even black) to bright-yellow. To determine the color transition point, the thermochromic films were dipped into ethylene glycol (a non-solvent liquid for polystyrene) and the temperature increased from room temperature to 200 °C, at a controlled rate of 1 °C/min. The transition point was simply determined by eyes. Figure 6 shows the reversible color variation of a polystyrene film containing dodecyl-thiolate capped silver nanoparticles. As visible, the nanocomposite color is brown at temperatures below 133 °C (left-side), and yellow above this temperature value (right-side). Prolonged thermal cycling of nanocomposite films does not significantly modify the original colorations.

The color transition temperatures found for nanocomposites based on silver nanoparticles capped by different types of thiolate molecules are given in Table 1. All samples showed color switching at a temperature depending on the precursor type. Only films based on polystyrene containing cyclohexyl-thiolate capped silver nanoparticles showed a stable yellow coloration. In particular, the temperatures of samples obtained by thermal decomposition of alkyl-thiolates with different chain lengths just slightly varied, whereas it was significantly shifted if one hydroxyl group was introduced in the capping thiolate molecule.

**Figure 7:**
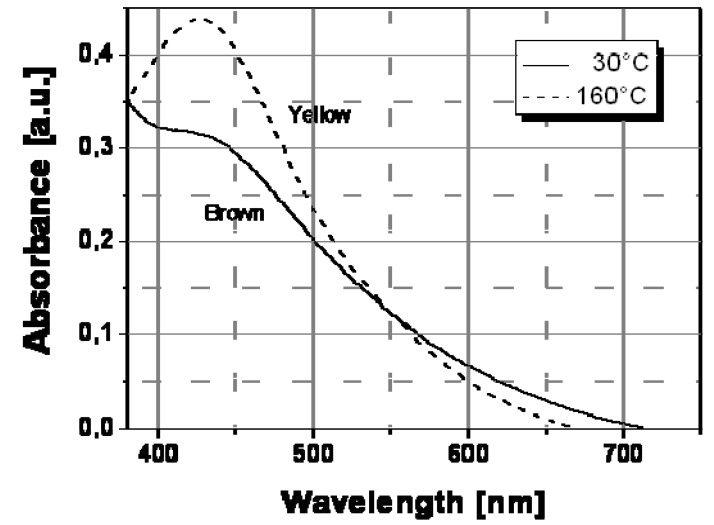
Absorption spectra recorded at two different temperatures for Ag/PS nanocomposite film obtained by thermal decomposition of silver dodecyl-thiolate in amorphous polystyrene.

The thermochromic effect was accurately investigated by spectroscopic characterization (UV-Vis absorption spectroscopy) of nanocomposite films at different temperatures. Figure 7 shows the spectral behavior of an Ag/PS film prepared by thermal decomposition of silver dodecyl-thiolate at 30 °C and 160 °C. As visible, there is a significant evolution of the absorption band with temperature. In particular, the extension of surface plasmon absorption band on the visible spectral region decreased substantially above a temperature of ca. 130 °C, while the corresponding intensity of the plasmon peak increased. Such spectral change may explain the observed color tuning from dark-brown to a dominant yellow with a temperature increase.

**Table 1:** Temperature values of color change and thermodynamic properties of reversible thermochromic films obtained by decomposing different silver precursors. The characteristics of the minor endo-thermal peak in the HO(CH_2_)_11_SAg thermogram are given in the brackets.

Thiolate	Temperature of color change (°C)	Interdigitation crystals melting range (°C)	ΔH (J/g)
CH_3_ (CH_2_)_11_SAg	133	116.5-138.0	1.652
CH_3_ (CH_2_)_15_SAg	129	119.5-140.7	2.962
CH_3_ (CH_2_)_17_SAg	123	115.0-138.5	2.865
HO(CH_2_)_11_SAg	162	133.0-170.1 (112.0-122.0)	1.895 (0.300)
C_6_H_5_SAg	-	-	-

**Figure 8:**
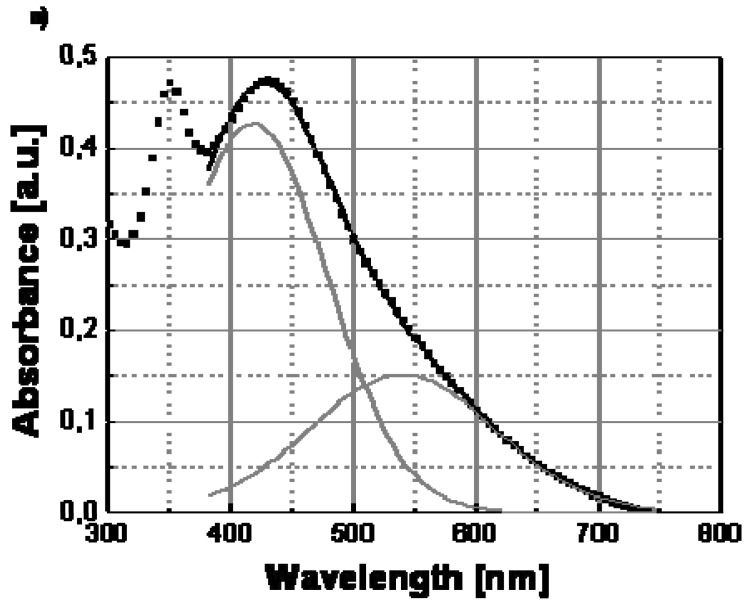

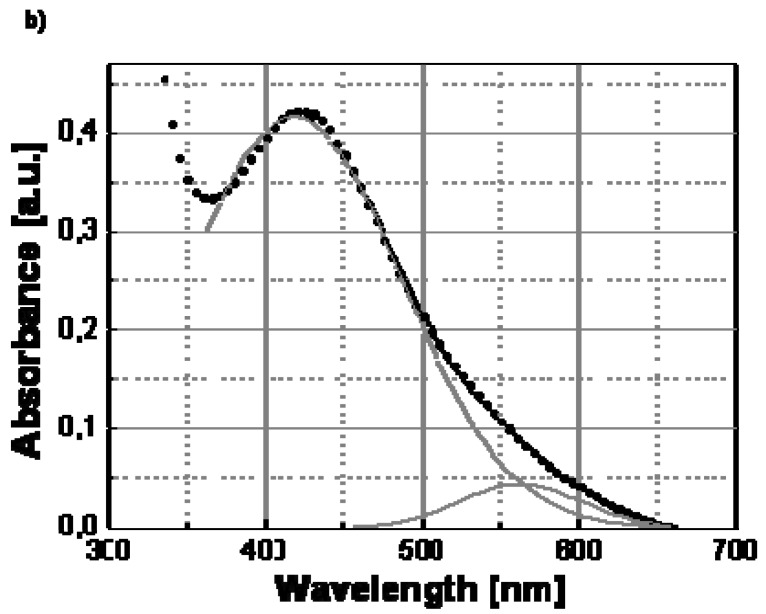
Deconvolution of the surface plasmon absorptionof Ag/PS nanocomposite obtained by thermal decomposition of silver dodecyl-thiolate at temperature below and above the transition temperature of 120 °C: (a) 30 °C; (b) 160 °C. The red curves are obtained in each case by adding the contributions of the Gaussian curves (grey lines) and smoothly interpolate the experimental data (square symbols).

To understand the spectral change in Figure 7, the transversal and longitudinal oscillation contributions to the spectra of Ag/PS film recorded a temperature lower and above the transition point should be compared. In particular, these two contributions to the silver nanoparticle surface plasmon resonance spectrum can be determined by convolving the full spectrum with two Gaussian functions (see Figure 8) centered at different wavelengths, the longer one corresponding to the longitudinal oscillations of the polarized nanoparticles, and the shorter one to the transversal oscillations. The two Gaussian curves are shown as continuous grey lines in Figure 8a and Figure 8b, respectively, for the two temperatures of 30 °C (below the transition temperature) and 160 °C (above the transition temperature).

The appearance of longitudinal oscillations is a well-known effect predicted in the framework of the Mie theory for aggregated nanoparticle structures [21]. While, the peak of the absorbance spectrum is basically determined by the transversal resonance of the isolated nanoparticles, the longitudinal oscillations at longer wavelengths produce a broadening of the spectrum. Both Figure 8a and Figure 8b show that the longitudinal contribution at longer wavelength (560 nm) is clearly lower than the transversal one at shorter wavelength (430 nm).

The color change should be related to a variation of the inter-particle distance at a special temperature and in particular above this temperature, nanoparticles do not anymore interact among themselves since they are located a higher distance and a substantial reduction of the longitudinal oscillations follows (see Figure 8b). Therefore, the longitudinal contribution to the absorbance spectrum is reduced at the higher temperature, according to the decrease of the corresponding convolution Gaussian peak compared to that visible in Figure 8a. As a result of the disappearance of the longitudinal oscillations we observe a spectral bandwidth decrease of ca. 100 nm and precisely from 750nm, at temperatures below the transition point, to ca. 650 nm, at temperatures above such transition point.

Optical spectra recorded for different times at temperatures above the color transition point have shown that these nanocomposite films are characterized by a very stable optical absorption during the time.

**Figure 9:**
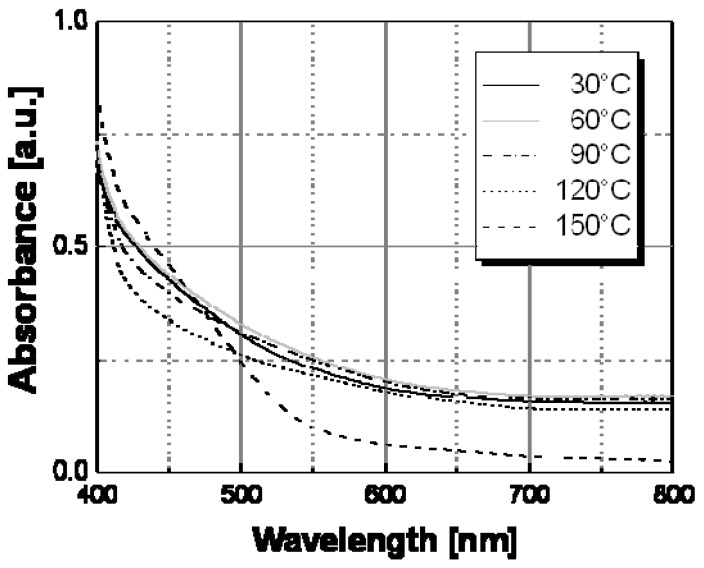
Absorption spectra at different temperatures of Ag/PS nanocomposite samples obtained by thermal decomposition of silver hexadecyl-thiolate in amorphous polystyrene.

**Figure 10:**
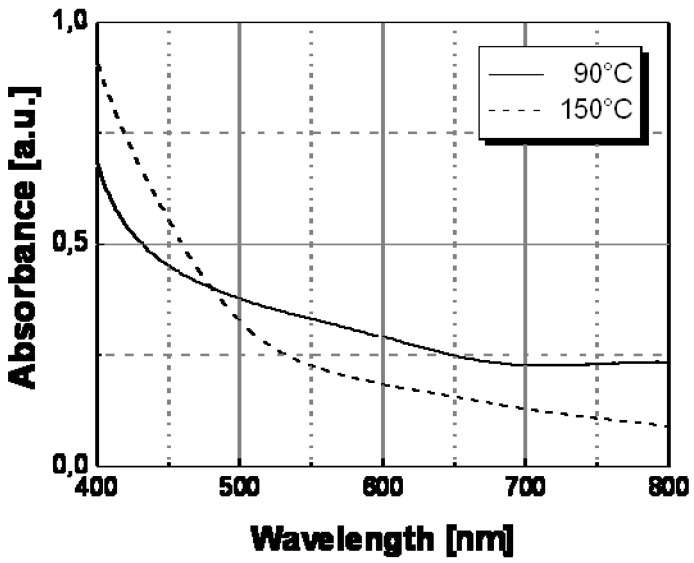
Absorption spectra recorded at two different temperatures for Ag/PS nanocomposite sample obtained by thermal decomposition of silver octadecyl-thiolate in amorphous polystyrene.

**Figure 11:**
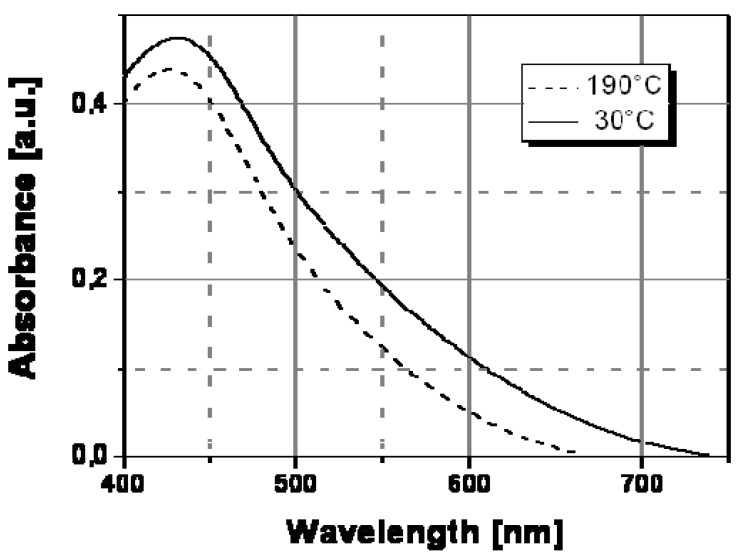
Absorption spectra recorded at two different temperatures for Ag/PS films obtained by thermal decomposition of HO(CH_2_)_11_SAg in amorphous polystyrene.

The optical behavior of nanocomposite films prepared by thermal decomposition of silver hexadecyl-thiolate (see Figure 9), silver octadecyl-thiolate (see Figure 10), and silver 11-hydroxy-1-undecylthiolate (see Figure 11) have been obtained in a similar way. All samples showed the same spectral behavior with temperature, consisting in a decrease of the contribute of the longitudinal oscillation mode above a certain temperature. Practically, a color change located at a temperature in the range 120 °C–160 °C was observed with all samples obtained by thermal decomposition of alkyl-thiolates of silver, and only the sample based on HO(CH_2_)_11_SAg in Table I showed a color transition located at a significantly higher temperature value (ca. 160 °C). The results of this spectroscopical analysis were confirmed by the simple visual determinations of the color switching temperatures.

In order to evidence the correlation between the color change and the melting of the interdigitated thiolate-capping crystallites, thermochromic films were analyzed by Differential Scanning Calorimetry (DSC). All thermograms included a first-order transition corresponding to the polystyrene glass transition temperature (Tg) at ca. 89 °C, followed by an endothermic peak. The extension of endo-thermal peaks and the corresponding enthalpy values are given in Table 1.

The endo-thermal signals visible in the DSC thermograms have been attributed to the melting of the crystallites generated by interdigitation of thiolate chains present on the surface of neighbor nanoparticles. As visible, the color transition points are located exactly in the temperature range of crystallite melting estimated by DSC, consequently the color switching should be related to the melting of interdigitated thiolate-capping crystallites and precisely it should be caused by the little change of the inter-particle distance caused by such melting. On the other hand, the presence of a melting point in the DSC thermogram confirms the presence of thiolate-capped silver nanoparticles inside the nanocomposite sample.

The obtained DSC results clearly suggest that the degree and the amount of interdigitation for simple normal alkyl-thiolate chains is only lightly affected by the number of methylene groups (i.e., -CH_2_-) present into the organic chain, and this implies that very similar color switching temperatures can be obtained with these samples.

The DSC-thermogram of a sample obtained by thermal decomposition of HO(CH_2_)_11_SAg shows two different endothermic peaks. The small peak at lower temperature corresponded to the melting point of -(CH_2_)_n_- present in the organic capping layer, whereas the second peak at higher temperature can be attributed to the collapse of the interdigitation between the ends of thiolate chain on neighbor nanonanoparticles. In this case, color switching temperature results much higher for the presence of -OH groups that produced stronger interaction in the interdigitation regions. Finally, the temperatures of color switching did not significantly change with the alkyl-thiolate length, but a significant change in the thiolate structure (e.g., the presence of one hydroxyl group) was able to affect the interdigitation crystals melting point.

**Figure 12:**
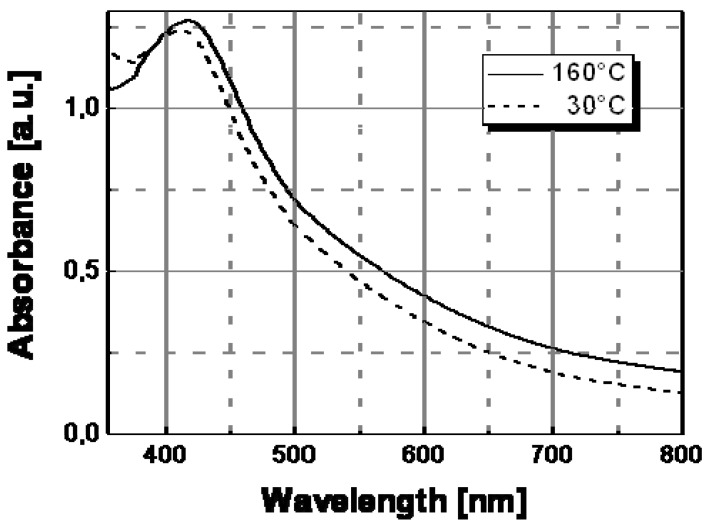
Dodecyl-thiolate capped silver nanoparticles embedded into epoxy, showing a stable surface plasmon coloration. The absence of thermochromism should be related to the rigid molecular structure which does not allow inter-particle distance modification.

A fundamental requirement for such a type of active optical devices is that the embedding optical medium may allow a volumetric change (expansion) to the hosted silver nanoparticle aggregates. For such a reason these devices must be exclusively based on linear polymers (thermoplastic polymers) and usually the color change is observed at a temperature higher than the polymer glass transition temperature (rubbery state). In particular, this type of thermochromism have been experimentally observed for thiolate-capped silver particles embedded into thermoplastic matrices, like polystyrene, poly(ethylene imines), poly(vinyl acetate), etc. On the contrary, as shown in Figure 12, aggregates of thiolate-capped silver nanoparticles embedded into a thermosetting matrix (for example, an epoxy resin) does not show thermochromism on heating but only a stable coloration. The absence of a change in the surface plasmon absorption for these materials should be related to the cross-linked structure characterizing these systems. In fact, in the present case particles are embedded into a three dimensional network with many transversal bonds (cross-links) that probably prevent particle movements.

**Figure 13:**
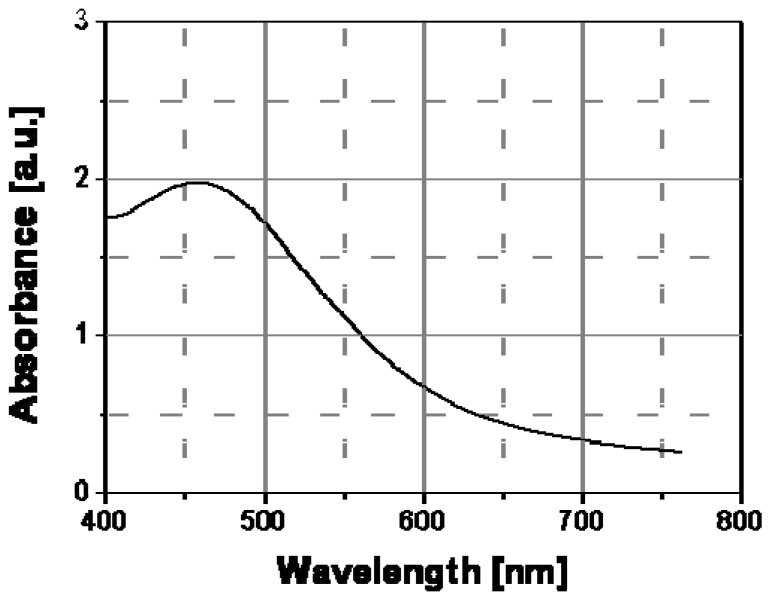
Optical absorption spectrum of cyclohexyl-thiolate capped silver nanoparticles embedded into an amorphous polystyrene matrix.

To observe the thermochromic phenomenon, also the presence of long chain capping thiolates on the silver nanoparticle surface is required. Films based on amorphous polystyrene embedding silver nanoparticles capped by short-chain thiolates molecules like cyclohexyl-thiolate were transparent and showed the characteristic yellow coloration, produced by the surface plasmon resonance of insulated silver nanoparticles (see Figure 13). The optical absorption did not change with temperature and the DSC-thermogram of annealed films did not show any thermodynamic transitions. The observed stable optical behavior should be related to the absence of interdigitation crystallites in these nanocomposite systems.

Thermochromic metal-polymer nanocomposites are technologically useful materials [25,26,27], because they can be used to measure temperature values much higher than those allowed to traditional thermochromic systems based on liquid crystals, that are usually lower than 80 °C. Consequently, these reversible thermochromic materials can be exploited in many high-temperature applications like overheating indicators, IR laser beam detectors, etc. However, it is important to control the color-switching temperature value, in order to have materials that can switch color at different temperatures. The color change is related to a thermodynamic transition (the melting point) of crystallites produced by interdigitation of alkyl chains in the organic layers coating the nanoparticle surface. Consequently, higher melting points of the organic layer, allows higher color-switching temperatures. This melting temperature is related to the type of non-bonding interactions in the interdigitated layer. Thus, the strength of non-bonding physical interactions among the thiolate chains plays a fundamental role in controlling the threshold for material thermochromism. In the case of linear alkyl-groups (i.e., dodecyl-, hexadecyl, and octadecyl) very weak physical interactions are involved between the methylene groups (precisely, dipole-dipole and Van der Waals interactions) and, according to the melting intervals given in Table 1, cohesion inside the crystals does not increase with the chain length increasing but slightly decreases (probably, the crystal lattices made by very long linear alkane chains may collapse easier than those made by shorter chains). In order to increase the melting point of interdigitation crystallites, a functional group able to produce much stronger physical interactions among the thiolate coatings is required. Hydroxyl functions (-OH) interact by hydrogen bridges which are very strong dipole-dipole interactions, and therefore the collapse of the resulting interdigitation crystallites is observed only at a much higher temperature. In fact, according to DSC-measurements, films made of amorphous polystyrene embedding silver nanoparticles capped by hydroxyl-undecyl thiolate-group (HO-(CH_2_)_12_-S-) showed a melting point of ca. 160 °C and a well-visible color transition from brown to yellow at the same temperature.

## 5. Conclusions

Dispersions of thiolate-capped silver nanoparticles in amorphous polystyrene have been produced by decomposing silver dodecyl-thiolate (AgSC_12_H_25_) molecules dissolved in the polymer at a temperature of ca. 200 °C. This nanocomposite material in form of film showed a prompt and completely reversible thermochromic effect, with strong chromatic variation from dark-brown or black, depending on the nanoparticles concentration, to a bright-yellow color at a temperature of 133 °C. The color switching temperature was coincident with the melting point of crystallites generated by the interdigitation of dodecyl-thiolate chains present on the metal nanoparticles surface (that was ranging from 116 °C to 138 °C, as estimated by DSC). Such color-switching temperature only slightly changed with increasing of the alkyl-thiolate chain length (hexadecyl- and octadecyl-thiolate molecules were used). However, when a functional group, HO-, able to give stronger physical interactions (hydrogen-bridge bonds) was introduced at alkyl-chain end (i.e., HO-(CH_2_)_11_-S-), both the interdigitation crystallites melting point and the nanocomposite film color switching temperature resulted significantly modified (ca. 162 °C). Therefore, the color switching temperature of these reversible thermochromic films can be controlled by modifying of structure of the thiolate-capping molecules present on the surface of the silver nanoparticles.

## References

[1] Wolf E.L. (2004). Nanophysics and Nanotechnology – An Introduction to Modern Concepts in Nanoscience.

[2] Poole C.P., Owens F.J. (2003). Introduction to Nanotechnology.

[3] Shmid G. (2004). Nanoparticles – From Theory to Application.

[4] De Jongh L.J. (1994). Physics and Chemistry of Metal Cluster Compounds.

[5] Klabunde K.J. (2001). Nanoscale Materials in Chemistry.

[6] Carotenuto G., Longo A., de Petrocellis L., de Nicola S., Repetto P., Perlo P., Ambrosio L. (2007). Synthesis of molecular gold clusters with luminescence properties by mercaptide thermolysis in polymer matrices. Int. J. Nanosci..

[7] Susha A.S., Ringler M., Ohlinger A., Paderi M., LiPira N., Carotenuto G., Rogach A.L., Feldmann J. (2008). Strongly luminescent films fabricated by thermolysis of gold-thiolate complexes in a polymer matrix. Chem. Mater..

[8] Longo A., Pepe G.P., Carotenuto G., Ruotolo A., DeNicola S., Belotelov V., Svesdin A.K. (2007). Optical emission studies in Au/Ag nanoclusters. Nanotechnology.

[9] Moser W.R. (1996). Advanced Catalysts and Nanostructured Materials: Modern Synthetic Methods.

[10] Miller J.S., Drillon M. (2002). Magnetism: Molecules to Materials III, Nanosized Magnetic Materials.

[11] Rosén A. (1998). A periodic table in three dimensions: A sightseeing tour in the nanometer world. Adv. Quantum Chem..

[12] Carotenuto G., Nicolais L. (2004). Metal-Polymer Nanocomposites.

[13] Carotenuto G., Nicolais L. (2003). Nanocomposites, Metal-Filled. Encyclopedia of Polymer Science and Technology.

[14] Caseri W. (2000). Nanocomposites of polymers and metals or semiconductors: hystorical background and optical properties. Macromol. Rapid Commun..

[15] Mayer A.B.R. (1998). Formation of noble metal nanoparticles within a polymeric matrix: Nanoparticle features and overall morphologies. Mater. Sci. Eng. C.

[16] Dirix Y., Bastiaansen C., Caseri W., Smith P. (1999). Oriented pearl-necklace arrays of metallic nanoparticles in polymers: A new route toward polarization-dependent color filters. Adv. Mater..

[17] Dirix Y., Bastiaansen C., Caseri W., Smith P. (1999). Preparation, structure and properties of uniaxially oriented polyethylene-silver nanocomposites. J. Mater. Sci..

[18] Whetten R.L., Khoury J.T., Alvarez M.M., Murthy S., Vezmar I., Wang Z.L., Stephens P.W., Cleveland C.L., Luedtke W.D., Landman U. (1996). Nanocrystal gold molecules. Adv. Mater..

[19] Wang Z.L., Harfenist S.A., Whetten R.L., Bentley J., Evans N.D. (1998). bundling and interdigitation of adsorbed thiolate groups in self-assembled nanocrystal superlattices. J. Phys. Chem. B.

[20] Carotenuto G., Marletta G., Nicolais L. (2001). Dependence of the order-disorder transition temperature of 1-octadecanethiol/silver system on the substrate size. J. Mater Sci. Lett..

[21] Kreibig U., Vollmer M., Toennies J.P. (1993). Optical Properties of Metal Cluster.

[22] Carotenuto G., Martorana B., Perlo P., Nicolais L. (2003). A universal method for the synthesis of metal and metal sulfide clusters embedded in polymer matrices. J. Mater. Chem..

[23] Carotenuto G., Nicolais L., Perlo P. (2006). Synthesis of polymer-embedded noble metal clusters by thermolysis of mercaptides dissolved in polymers. Polym. Eng. Sci..

[24] Conte P., Carotenuto G., Piccolo A., Perlo P., Nicolais L. (2007). NMR-investigation of the mechanism of silver mercaptide thermolysis in amorphous polystyrene. J. Mater. Chem..

[25] Carotenuto G., LaPeruta G., Nicolais L. (2006). Thermo-chromic materials based on polymer-embedded silver clusters. Sens. Actuators B.

[26] Liu Y., Mills E.N., Composto R.J. (2009). Tuning optical properties of gold nanorods in polymer films through thermal reshaping. J. Mater. Chem..

[27] Tollan C.M., Marcilla R., Pomposo J.A., Rodriguez J., Aizpurua J., Molina J., Mecerreyes D. (2009). Irreversible thermochromic behavior in gold and silver nanorod/polymeric ionic liquid nanocomposite films. ACS Appl. Mater. Interfaces.

